# Painless Buccal Abscess With Intraoral Fistula in an Older Woman

**DOI:** 10.1016/j.acepjo.2026.100420

**Published:** 2026-05-26

**Authors:** Kyoko Yamamoto, Yoshihiro Aoki, Hiromasa Harada, Kensuke Takahashi, Shuhei Yamano, Koichi Hayakawa, Osamu Tasaki

**Affiliations:** 1 Department of Emergency Medicine, Nagasaki Harbor Medical Center, Nagasaki, Japan; 2 Coordination Office for Emergency Medicine and International Response, Acute and Critical Care Center, Nagasaki University Hospital, Nagasaki, Japan; 3 Acute and Critical Care Center, Nagasaki University Hospital, Nagasaki, Japan

**Keywords:** Actinomycosis, abscess, fistula, mouth, anaerobic bacteria, incidental finding

## 1 Case Presentation

**Figure 1:**
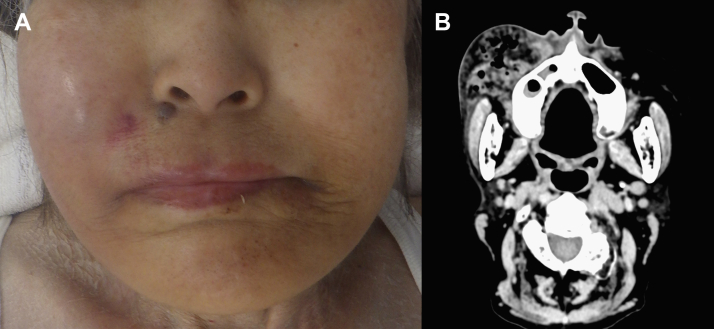
Right buccal subcutaneous abscess. (A) Clinical photograph demonstrating diffuse swelling of the right cheek with overlying erythema. (B) Axial computed tomography demonstrating a subcutaneous abscess with gas formation in the right buccal region.

**Figure 2:**
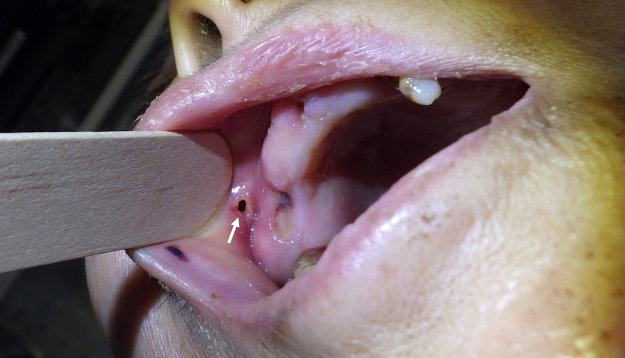
Intraoral fistula. Intraoral photograph showing a fistula on the right buccal mucosa (arrow) with spontaneous purulent drainage.

A woman in her 70s was brought to the emergency department with a right distal femoral fracture after being found immobile at home for an estimated 6 days. Her medical history included common bile duct stones without immunosuppressive conditions. On admission, she was alert and oriented, tachycardic with a normal respiratory rate, low-grade fever of 37.4°C, and no hypoxemia. Right cheek swelling with erythema was incidentally noted; the patient denied any associated pain (Fig 1A). Laboratory studies revealed leukocytosis, elevated C-reactive protein levels, and acute kidney injury. Computed tomography demonstrated a gas-containing subcutaneous abscess in the right buccal region (Fig 1B). Intraoral examination revealed poor oral hygiene and a fistula on the right buccal mucosa with purulent drainage (Fig 2). Aspirated material was submitted for anaerobic culture.

## 2 Diagnosis

### 2.1 Cervicofacial Actinomycosis Presenting as Buccal Subcutaneous Abscess With Intraoral Fistula

Anaerobic cultures yielded *Actinomyces* species, *Bacteroides thetaiotaomicron*, *Streptococcus anginosus*, and *Proteus mirabilis*. A dental origin was suspected, although the fistula opened onto the buccal mucosa rather than the gingiva. The painless, indolent presentation with fistula formation was characteristic of cervicofacial actinomycosis.^1^ The patient was treated with incision and drainage followed by intravenous ampicillin-sulbactam, transitioning to oral amoxicillin for a planned 6-month course. The diagnosis was based on isolation of *Actinomyces* species from the abscess, with co-isolated organisms interpreted as copathogens typical of polymicrobial actinomycosis.^1^^,^^2^ Although histopathological confirmation and acid-fast staining were not obtained, the clinical course and culture findings supported the diagnosis.

## Funding and Support

By *JACEP Open* policy, all authors are required to disclose any and all commercial, financial, and other relationships in any way related to the subject of this article as per ICMJE conflict of interest guidelines (see www.icmje.org). The authors have stated that no such relationships exist.

## Conflict of Interest

All authors have affirmed they have no conflicts of interest to declare.

## References

[1] Wong V.K., Turmezei T.D., Weston V.C. (2011). Actinomycosis. BMJ.

[2] Valour F., Sénéchal A., Dupieux C. (2014). Actinomycosis: etiology, clinical features, diagnosis, treatment, and management. Infect Drug Resist.

